# Black Soldier Fly Larvae Meals With and Without Stickwater Highly Utilized in Freshwater by Atlantic salmon (*Salmo salar*) Parr

**DOI:** 10.1155/anu/8827164

**Published:** 2025-05-06

**Authors:** André S. Bogevik, Erika Hanson, Tor Andreas Samuelsen, Katerina Kousoulaki

**Affiliations:** ^1^Department of Nutrition and Feed Technology, Nofima 5141, Fyllingsdalen, Norway; ^2^Department of Animal and Aquaculture Sciences, NMBU 1432, Ås, Norway

**Keywords:** aquafeeds, digestion, insects, minerals, processing, utilization

## Abstract

Black soldier fly larvae (BSFL) meal is a promising sustainable protein source for aquafeeds. Processing BSFL into meal and oil can be based on wet rendering technology where the raw material is heat treated and mechanically separated into press cake, stickwater (SW), and oil. In this study, to verify the effect of SW reincorporation into the press cake, dried BSFL cake and SW meal were included in feed mixes before extrusion. Four experimental feeds were prepared, containing 100 g kg^−1^ BSFL meal with a cake/SW ratio of 100/0, 90/10, 80/20 and 60/40 g kg^−1^, respectively and compared to a control feed in a trial with Atlantic salmon parr reared in freshwater. The feeds had similar nutritional value and all feeds were highly digestible. The highest content of manganese (Mn) was found in the BSFL cake feed (120 mgkg^−1^), was reduced with increased SW inclusion and lowest in the control feed. There were no dietary differences in growth or welfare with increased SW inclusion, and no negative impact of high dietary Mn levels. The high Mn content was not accumulated in the fish, and only resulted in an increased excretion of Mn. Further studies are needed to verify these results in Atlantic salmon postsmolt.

## 1. Introduction

Future growth in Norwegian aquaculture should be based on feed developed from more sustainable raw material sources by for example reduced use of freshwater and land resources per unit of production, and energy efficient processing into ingredients [1, 2]. Black soldier fly larvae (BSFL) are an example of such a source because they can convert by-products into high-quality protein [3]. Growing BSFL does not require large land areas, they have a short lifecycle and can be harvested frequently and year-round [4]. BSFL has successfully been used in diets for Atlantic salmon (*Salmo salar*) replacing 100% FM with promising results in tank experiments [5, 6] and at low inclusion levels (5%–10%) in sea cage experiments at commercial farms [7, 8]. However, variations in the nutrient digestibility of BSFL meal products are observed, with batch-to-batch variation of digestible protein from 85 to 92% in rainbow trout (*Oncorhynchus mykiss*) [9]. Furthermore, the needs for manganese (Mn) in insects [10], and accumulation to a high content in BSFL meal [11, 12] may limit the inclusion level in Atlantic salmon feeds. The European Union has set an upper limit of 100 mg kg^−1^ of Mn in aquafeeds (EU Regulation 2017/893/EC). Supplementation of Mn through mineral premixes is commonly needed in diets to meet the minimum requirement to maintain whole-body saturation of the mineral in Atlantic salmon parr (12–15 mg kg^−1^ feed) and smolt (26–34 mg kg^−1^ feed) [13, 14]. Deficiency of Mn results in reduced growth and skeletal abnormalities, while toxic levels of Mn cause disrupted homeostasis of other minerals in the fish [15, 16]. The inclusion of BSFL meal in the feeds may reduce the need of Mn supplementation to meet the requirement of the minerals in feeds to Atlantic salmon.

There are different routes for processing BSFL into dry products. This can be drying of whole larvae into a full-fat meal or producing a defatted BSFL meal by removal of oil from the dry larvae using a screw press at high temperature and pressure. However, the drying methods and high temperature may affect the nutritional quality of the meal [17, 18]), and reduce protein digestibility, and micronutrient content of for example vitamins [19]. Improved nutritional quality of the product may be achieved by the use of wet rendering comparable to what is used in fish meal (FM) processing. In this process, the wet BSFL raw material is heat treated, wet milled, and separated into a solid (cake), stickwater (SW), and oil fraction in a 3-phase decanter centrifuge [20]. A common practice in FM production is to concentrate the SW fraction in an evaporation stage from approximately 6%–9% to 30%–50% DM and add it back to the cake before drying and milling [21]. The SW fraction constitutes 20%–25% DM of the total FM in a “whole meal” and is added back to increase the yield and reduce losses in the wastewater system. The SW consists mainly of WSPs and other highly valuable macro and micronutrients, and it is documented that these compounds have positive effects on the feed intake and performance in Atlantic salmon [22, 23, 24, 25]. The SW fraction has also a positive plasticization effect improving the physical quality of extruded feed [26]. The SW inclusion in BSFL meal production will increase investment costs for evaporators and piping and increase space requirements and dryer load. However, reincorporating SW in the BSFL meal will increase the yield and are hypothesized to have positive physiological effects on the fish, as documented for SW in FM. It is therefore important to improve our knowledge regarding the chemical composition of SW and investigate the potential benefits of adding BSFL SW back to the BSFL meal in diets for salmon. The objectives for this study were to explore the effect of increased BSFL meal Mn content on accumulation in salmon tissues, and the impact of BSFL SW inclusion level in feeds on nutrient digestibility, growth performance, and welfare of freshwater Atlantic salmon parr.

## 2. Materials and Methods

The BSFL meal used in the present study was produced noncommercially by Innovafeed in Paris (France), under the scope of the Millennial salmon project (Grant number: 319987, funded by the Research Council of Norway and the industrial partners INNOVAFEED, Corbion, Cargill, Auchan, MOWI and Labeyrie fine foods). The BSFL were raised on a diet consisting mainly of wheat bran. The BSFL cake meal and SW powder were produced based on wet rendering technology and the cake and SW fraction dried separately. The dried BSFL cake and SW were shipped to Nofima's Aquafeed technology centre (www.aquafeed.science, Bergen, Norway) for inclusion in feeds for Atlantic salmon. A control feed, which contained neither BSFL cake, nor SW, was formulated to adapt the fish size in the trial and based on ingredients used by the Norwegian salmon feed producers in 2020 [2]. In addition, four experimental feeds containing 100 g kg^−1^ BSFL meal with a cake/SW ratio of 100/0 (BFSL-cake), 90/10 (SW-1), 80/20 (SW-2) and 60/40 g kg^−1^ (SW-3) were produced. The feeds were balanced with essential AAs in crystalline form, vitamins, minerals, and phospholipids to meet the salmon parr nutrient requirements (Table 1). The feeds were produced using a combined preconditioner and corotating twin-screw extruder system (TX-52, Wenger Manufacturing Inc., Sabetha, KS, The United States of America) and dried in a horizontal conveyor belt dryer with two drying chambers (Mitchell Dryers, CAD Works Engineering Ltd, Carlisle, UK). The feeds were thereafter coated with oil using a vacuum coater (PG-60VC, Dinnissen BV, Sevenum, The Netherlands). The produced pellets had a diameter of 2.5 mm.

The fish trial was conducted at Nofima Research Station for Sustainable Aquaculture (Sunndalsøra, Norway) in a flow-through aquaculture system. The five diets were fed over a period of 8 weeks to triplicate tanks (150 L) 100 Atlantic salmon (*S. salar*) parr (start weight 25.7 ± 0.1 g) in freshwater. Water temperature was held at 12°C with 106% oxygen saturation. The fish were fed every 15 min using automatic feeders. At the termination of the trial 10 fish per tank were euthanized with Finquel MS-222 (20 g L^−1^; tricaine methane sulfonate, Scan-Vacc, Hvam, Norway). Fish body weight, standard length, liver weight, and external welfare score were recorded as described by Noble et al. [27]. The FISHWELL welfare indicators system score fin and skin damage from value zero (normal) to three (no remaining fin(s) and severe scale loss). Hindgut content, liver, midgut, and muscle tissue were collected from 10 fish per tank and stored at −20°C before freeze-drying and chemical analysis. The midgut was cut diagonally and rinsed with PBS-solution (VWR, Ohio, USA), and muscle was sampled from the left NQC area after fileting and removal of the skin. All samples were pooled per tank. Five fish per tank were collected for whole-body analyses. For this, the fish were cut into smaller parts and homogenized using a meat grinder and a subsample was freeze-dried before chemical analysis. In addition, 10 fish per tank were collected and stored at −20°C before being x-rayed for skeletal deformities using an IMS Giotto mammography system (model number 6020/3, IMS Giotto, Bologna, Italy). The remaining fish in each tank were bulk weighed, stripped [28], and the collected feces were pooled together with hindgut content collected from the 10 fish sampled for tissues and stored at −20°C before freeze-drying and chemical analysis.

Total fat [29] and protein (Kjeldahl method; ISO 5983-1997) were analyzed by the accredited laboratory Nofima BioLab, (Bergen, Norway). Water-soluble protein (WSP) and peptide size distribution were analyzed as described in Bogevik et al. [30]. For total amino acid (AA) profile determination, samples were hydrolyzed in 6 M HCl for 22 h at 110°C and analyzed by HPLC using a fluorescence technique for detection [31]. The total levels of tryptophan and cysteine in the diets were not determined. FAAs, taurine, and anserine were analyzed similarly, as described by Bidlingmeyer et al. [32]. Minerals were analyzed as described in Kokkali et al. [33] with sample preparation in a single reaction chamber oven (UltraWave, Milestone, Sorisole, Italy) and element determination in an Inductively Coupled Plasma Optical Emission Spectroscopy (ICP-OES) (Agilent 5110 VDV, Agilent Technologies, Mulgrave, Australia). Due to the high mineral content in the feces, samples were diluted twice as much compared to the other samples before element analysis. All samples were run in duplicates.

All calculation were performed in Microsoft Excel. The specific growth rate was calculated as ((ln(final weight) − ln(start weight))/days in trial) × 100, and the condition factor was calculated as body weight divided by length3 × 100. Apparent digestibility coefficient (ADC) of nutrients and energy in the test feeds was calculated from the following formula: ADC=100 − 100 × [*Yd*/*Yf*] × [*Nf*/*Nd*] where *d* is feed, *f* is feces, *Y* yttrium content and *N* nutrient content. All statistical analyses were performed using Statistica 14 software for Windows (StatSoft Inc., Tulsa, USA). The effects of diets on fish performance and chemical analysis between dietary groups were subjected to General Linear model analysis using one-way ANOVA for detection of significant differences (*p*  < 0.05), followed by the Tukey post hoc test.

## 3. Results and Discussion

BSFL meal is a promising circular economy-based protein source for Atlantic salmon feeds [5, 6, 11]. In the present study, BSFL were separated in press cake and SW to study the physiological effects of the two fractions, solids, and water-soluble biomass, in farmed Atlantic salmon. The chemical composition of the BSFL fractions and produced test feeds is shown in Table 2. The dried BSFL cake and SW had a protein content of 61.3 and 50.0% respectively, whereas the protein in the SW meal was composed exclusively of WSP. There was a lower content of Ca and micro elements in the BSFL SW, including a 10-fold lower Mn concentration compared to the BSFL press cake meal. The compositional differences in the two insect meal fractions were reflected in the experimental diets, showing increasing content of WSP and decreased content of Mn with increasing dietary inclusion level of BSFL SW to the expense of the press cake meal. The control feed had similar WSP content compared to the feed with the highest content of BSFL SW (40 g kg^−1^), while the dietary content of Mn was lowest (60 mg kg^−1^) in the control and highest (120 mg kg^−1^) in the diet with 100 g kg^−1^ BSFL cake and no inclusion of BSFL SW. The total and free amino acid (FAA) composition of the experimental diets is shown in Supporting information: Tables S1 and S2, respectively. In that respect, we observed only minor differences between the experimental diets, which are explained by the respective raw material composition. The BSFL-based feeds with increasing SW levels had respectively increasing levels of the nonessential amino glutamic acid and proline and decreasing levels of lysine as compared to feeds with no SW inclusion. Larger relative differences were seen in the FAA profile of the experimental diets, with the control diet containing higher total levels of FAAs (3.1%) and more of the characteristic for marine ingredients FAAs creatinine (41% more) and taurine (52% more), as compared to the diets added the BSFL meals showing variation in FAA content not correlated to the BSFL SW inclusion (2.6–2.9%). The increased content of WSP by BSFL SW inclusion was mainly in the form of small (<200 Dalton) to intermediate-sized peptides (200–10 000 Dalton; Table 2), known to improve growth in Atlantic salmon postsmolt [25].

We observed no statistically significant differences in growth with increasing the WSP level from 7.5% (BSFL-cake diet) to 9% (control and SW 3 diet) in the present trial. Atlantic salmon grew well during the feeding trial, from a starting body weight of 25.7 ± 0.1 to a final body weight of 79.3 ± 3.7 g, when fed the test feeds containing 100 g kg^−1^ BSFL meal with different inclusions of SW. The performance results from the feeding trial are summarized in Table 3. We observed no statistically significant differences between the dietary groups on final body weight or growth rate (one-way ANOVA; *p*  > 0.05) and with a specific growth ratio (SGR) between 1.9 and 2.0. The higher content of dietary FAAs following the water-soluble fraction of marine raw materials have a known attractant function for carnivorous fish, such as, proline, L-*α*-amino-*β*-guanidinopropionic acid, leucine, and phenylalanine in the case of salmonids [34, 35]. These are overrepresented in ingredients such as fish and krill water solubles, resulting in increased feed intake rates in European sea bass (e.g., [36]) and farmed Atlantic salmon (e.g., [37]). Likewise, free glycine is shown to enhance feed intake rates in chinook salmon [38]. In our study, there was a significant correlation between final body weight and the ratio between essential and nonessential dietary AAs (*R*^2^=0.9227; *p*  < 0.01) but not the total essential dietary AAs (*R*^2^=0.127; *p*  > 0.10). The lower levels of essential FAAs and essential/nonessential AA ratio in BSFL meal can easily be compensated for by supplementation of for instance Met and Lys in crystalline form or combining appropriate in that respect dietary ingredients, to reduce losses in dietary AA digestibility, protein synthesis, and fish body growth [11]. Further measurements of the fish revealed similar condition factor, liver size, and slaughter yield between dietary treatments. Welfare measures such as scale loss, dorsal fin damage, and pectoral fin damage were recorded, again without observing any significant differences between dietary treatments. Skeletal deformities were recorded as well. There were few cases with skeletal deformities, and the deformities that were found were mainly vertebral fusions in the spine. There were more cases of skeletal deformities in the control treatment, though not significantly different as compared to the BSFL test treatments (ANOVA; *p*  > 0.05).

**Figure 1:**
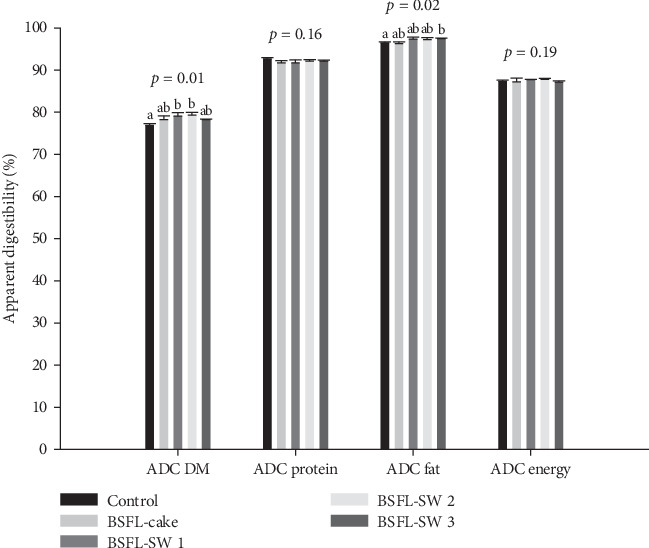
Digestibility of lipid, protein, and dry matter between dietary groups. Mean ± SD, *n* = 3 tanks. Statistics are determined by one-way ANOVA (*p*  < 0.05), followed by Tukey post hoc showing differences between diets by superscript letters.

The feeds used in the present trial had similar apparent digestibility (ADC) coefficient of protein and energy, while small differences were observed in the ADC of dry matter (DM) and total fat between the dietary groups of salmon parr (Figure 1). The control group showed a significantly lower DM ADC compared to salmon fed the feeds with low inclusion of BSFL SW (10 and 20 g kg^−1^) and lower total fat digestibility compared to salmon fed the highest content of BSFL SW (40 g kg^−1^). In our study, ~15% of the dietary protein in the test diets originated from the BSFL meals, substituting about 35% of the dietary FM protein. Weththasinghe et al. [39] found lower digestibility of fat and reduced growth in salmon parr (start weight 34 g) fed feeds where 25% of the dietary protein was replaced with BSFL meal (323 g kg^−1^ of the diet). Nevertheless, in agreement with the inclusion level of BSFL in our study, the authors showed minor differences in ADC and no differences in growth when only 6.25% and 12.5% of the dietary protein was replaced with BSFL meal compared to the control. Though, feed intake could not be measured in our study due to the small pellet size used, the numerically lower body weight growth and improved ADC results using BSFL meals in the diets, may be related to marginally reduced feed intake rates in the BSFL groups as compared to the FM based control.

**Figure 2:**
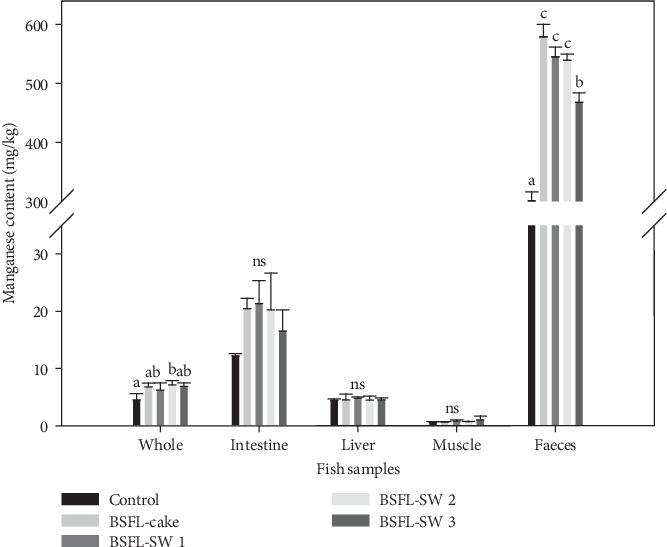
Manganese content in different tissue samples. Mean ± SD, *n* = 3 tanks. Statistics are determined by one-way ANOVA (*p*  < 0.05), followed by Tukey post hoc showing differences between diets by superscript letters.

In the present study, inclusion of 100 g kg^−1^ BSFL cake meal resulted in a dietary Mn content of 120 mg kg^−1^, according to EU Regulation (2017/893/EC), above the upper limit of Mn (100 mg kg^−1^) in commercial feed. Increased inclusion of BSFL SW reduced the Mn content within this limit. Thus, dietary Mn supplementation in commercial feeds will be unnecessary using BSFL meal levels comparable to this study. The results of the present study document that excess Mn is not absorbed or accumulated in salmon tissues, but increases the Mn level secreted through the feces (Figure 2) seen by significantly less Mn in feces from the control group compared to feces from fish fed the BSFL meal rich diets (ANOVA; *p*  < 0.05). Furthermore, significantly lower Mn content was analyzed in feces from salmon fed with the highest inclusion of BSFL SW compared to the other BSFL feeds. The lowest Mn content was found in the muscle and highest in the midgut tissue but with no dietary treatment differences within the different fish tissues. The midgut tissues were scraped clean for feces and rinsed in PBS solution before analysis. Midgut samples are in direct contact with feces, and a higher Mn content was expected compared to the other tissue samples. In extreme cases, when dietary intake of Mn is too high, fish adapt by reducing intestinal absorption, enhancing liver metabolism, and increasing excretion through the bile and feces [14]. Absorbed Mn is transported through the blood to the liver before being stored in the bones [14, 40]. The whole fish samples revealed however no differences in Mn content between the dietary groups, and there were no significant differences in welfare indicators, such as scale loss, pectoral fin damage, dorsal fin damage or skeletal deformities in the high Mn content BSFL cake fed fish compared to fish from the other treatments. At a toxic level of Mn homeostasis of other minerals is affected, for example lower absorption and reduced whole-body content [15]. Other minerals analyzed in tissues and feces showed only minor differences between dietary groups, mainly reflecting dietary composition by increased fecal secretion at a higher content of calcium in the control diets compared to the BSFL diets (Supporting Information: Table S3). One exception was significantly higher whole-body content (*p* < 0.05) and a tendency of higher liver content (*p*=0.077) of copper in the control group compared to the BSFL not reflecting the feed content that was similar between the dietary groups. However, there are no other indications that the Mn content of the diets may have affected whole-body saturation of other minerals in the present study that could impacted the welfare of the fish.

## 4. Conclusion

Atlantic salmon parr grows well with no indications of reduced performance, welfare scores or changes in whole-body and tissue composition fed diets containing 10% BSFL with or without SW addition. Attention should be paid to balancing diets containing BSFL meals to account for a lower ratio of essential/nonessential AAs and FAAs. BSFL meal contains high levels of the essential trace mineral Mn. BSFL SW contains less Mn compared to BSFL cake, so combining these will give a product with lower Mn content. The high inclusion of BSFL meal will result in dietary Mn content above the legal limits for aquafeed which would render additional Mn supplementation in mineral premixes illegal. Moreover, we saw no evidence of whole-body or tissue (muscle, liver, and midgut) oversaturation in dietary Mn in salmon parr when fed diets with increasing levels of Mn. On the contrary, the fish given diets with excess Mn levels could effectively excrete those through their feces. No negative effects in salmon performance or tissue mineralization for other essential microminerals when fed diets containing up to 120 mg/kg Mn were observed in this study. Further studies are needed to verify these results in Atlantic salmon postsmolt, especially related to the positive effects observed by SW inclusion in FM on the growth of salmon in seawater.

## Figures and Tables

**Table 1:** Diet composition by ingredients (g kg^−1^).

	Control	BSFL-cake	SW 1	SW 2	SW 3
Fish meal^1^	250.0	150.0	150.0	150.0	150.0
SPC^2^	150.0	150.0	150.0	150.0	150.0
Wheat gluten^3^	140.0	160.0	160.0	160.0	160.0
Faba beans^4^	80.0	80.0	80.0	80.0	80.0
Wheat^5^	156.9	136.9	136.9	136.9	136.9
BSFL cake^6^	—	100.0	90.0	80.0	60.0
BSFL stickwater^6^	—	—	10.0	20.0	40.0
Fish oil^1^	120.0	120.0	120.0	120.0	120.0
Rapeseed oil^7^	30.0	40.0	40.0	39.0	38.0
Linseed oil^8^	10.0	—	—	1.0	2.0
Lecithin from rapeseed^9^	9.5	—	—	—	—
Marin lecithin^10^	0.5	10.0	10.0	10.0	10.0
L-Threonine^11^	4.9	4.9	4.9	4.9	4.9
L-Lysine^11^	9.4	9.4	9.4	9.4	9.4
Methionine^11^	4.2	4.2	4.2	4.2	4.2
Histidine^11^	1.5	1.5	1.5	1.5	1.5
Mineral premix^11^	5.0	5.0	5.0	5.0	5.0
Vitamin premix^11^	5.0	5.0	5.0	5.0	5.0
Vitamin C^11^	0.5	0.5	0.5	0.5	0.5
Choline chloride^12^	2.0	2.0	2.0	2.0	2.0
Monosodium phosphate^11^	20.0	20.0	20.0	20.0	20.0
Carop. Pink^13^	0.5	0.5	0.5	0.5	0.5
Yttrium oxide^14^	0.1	0.1	0.1	0.1	0.1

*Note:* Ingredients purchased from ^1^Pelagia (Egersund, Norway), ^2^CJ Selecta (Distrito, Brazil), ^3^Roquette (Lestrem, France), ^4^Soufflet (Nogent-sur-Seine, France), ^5^Norgesmøllene AS (Vaksdal, Norway), ^6^Innovafeed (Paris, France), ^7^Emmelev (Otterup, Denmark), ^8^Vandeputte (Mouscron, Belgium), ^9^Marvesa (Den Haag, The Netherlands), ^10^TripleNine (Esbjerg, Denmark), ^11^Vilomix (Hønefoss, Norway), ^12^Berg+Schmidt (Hamburg, Germany), ^13^ DSM, firmenich (Basel, Switzerland), ^14^VWR (Oslo, Norway).

**Table 2:** Nutritional content of BSFL cake and SW, and experimental feeds.

Analysis	Ingredients		Feeds				
	BSFL-SW	BSFL-cake	Control	BSFL-cake	SW 1	SW 2	SW 3
Total dry matter (%)	95.7	97.8	92.3	91.7	91.4	91.7	91.6
Crude protein (%)	50.0	61.3	43.5	44.0	43.7	43.9	44.2
Total fat (%)	9.8	10.7	22.4	23.0	22.9	22.1	22.1
WSP (%)	50.3	<5.0	8.86	7.48	7.95	8.02	9.12
Peptide size (daltons; %)^a^							
MW > 10,000	1.8	5.1	14.4	17.7	17.4	15.2	14.5
MW = 10,000–200	56.5	51.6	35.7	35.1	36.1	38.4	40.1
MW < 200	41.7	43.5	49.9	47.2	46.5	46.4	45.3
Minerals (mg/kg)							
Ca	3120	13 100	8600	7000	6800	6800	6600
Mg	4610	4380	2300	2200	2100	2100	2100
Cu	5	15	17	17	15	15	15
Fe	63	207	250	240	240	250	250
Mn	49	584	60	120	110	110	94
Zn	53	240	180	180	170	170	170

^a^Peptide size distribution for different molecular weights (MW) in water-soluble protein (WSP) fraction.

**Table 3:** Mean, standard deviation and *p*-Value for start weight, final weight, specific growth rate (SGR), thermal growth coefficient (TGC), condition factor (CF), hepatosomatic index (HSI), visceral somatic index (VSI), yield %, welfare indicators such as scale loss, dorsal fin damage, pectoral fin damage, and skeletal deformities.

	Control	BSFL cake	Cake + SW 1	Cake + SW 2	Cake + SW 3	*p*-Value
Start weight (g)	25.6 ± 0.2	25.8 ± 0.1	25.8 ± 0.1	25.8 ± 0.1	25.7 ± 0.1	0.103
Final weight (g)	81.7 ± 3.8	78.8 ± 3.2	79.1 ± 5.9	79.3 ± 1.4	77.4 ± 4.6	0.267
SGR	2.0 ± 0.1	2.0 ± 0.1	2.0 ± 0.1	2.0 ± 0.0	1.9 ± 0.1	0.722
TGC	1.8 ± 0.1	1.7 ± 0.1	1.7 ± 0.1	1.7 ± 0.0	1.7 ± 0.1	0.741
K-factor	1.5 ± 0.1	1.5 ± 0.1	1.5 ± 0.1	1.5 ± 0.1	1.5 ± 0.1	0.591
HSI	0.1 ± 0.0	0.1 ± 0.0	0.1 ± 0.0	0.1 ± 0.0	0.1 ± 0.0	0.757
VSI	12.9 ± 1.8	12.9 ± 1.2	13.2 ± 1.0	13.1 ± 1.2	13.1 ± 1.1	0.821
Yield	87.1 ± 1.8	87.1 ± 0.9	86.8 ± 1.0	86.9 ± 1.2	86.9 ± 1.1	0.458
Welfare score (scale 1–3)	—	—	—	—	—	—
Scale loss	1.1 ± 0.3	1.4 ± 0.5	1.0 ± 0.2	1.1 ± 0.3	1.2 ± 0.4	0.355
Fin damage	1.5 ± 0.6	1.5 ± 0.7	1.7 ± 0.6	1.5 ± 0.6	1.4 ± 0.6	0.663
Skeletal deformities	0.3 ± 0.1	0.1 ± 0.2	0.2 ± 0.3	0.0 ± 0.0	0.1 ± 0.1	0.226

*Note*: Mean ± SD; *n* = 3. Statistics by one-way ANOVA, dietary differences at *p*  < 0.05.

## Data Availability

The data that support the findings of this study are available from the corresponding author upon reasonable request.

## Supporting Information

Supporting Information Table S1. Total amino acid (AA) composition in different diets, and AA content of BSFL press cake, BSFL SW and fish meal used in different diets expressed in g 100 g^−1^ feed. Table S2. Free amino acid (FAA) composition of the experimental diets used in the present study expressed in g 100 g^−1^. Table S3. Mineral content in fish feces and whole-body and tissue mineralization in salmon fed diets without or with BSFL meal supplementation with graded levels of SW reincorporation. Values are in mg kg^−1^ mean (*N* = 3 tanks) ± pooled standard error of mean (SEM).
