# Supplementary material for: Black Soldier Fly Larvae Meals With and Without Stickwater Highly Utilized in Freshwater by Atlantic salmon (Salmo salar) Parr
Source: Aquac Nutr. 2025 May 6;2025:8827164. doi: 10.1155/anu/8827164 (PMC12074852; doi:10.1155/anu/8827164)
Supplement: Supporting Information — Table S1. Total amino acid (AA) composition in different diets, and AA content of BSFL press cake, BSFL SW and fish meal used in different diets expressed in g 100 g−1 feed. Table S2. Free amino acid (FAA) composition of the experimental diets used in the present study expressed in g 100 g−1. Table S3. Mineral content in fish feces and whole-body and tissue mineralization in salmon fed diets without or with BSFL meal supplementation with graded levels of SW reincorporation. Values are in mg kg−1 mean (N = 3 tanks) ± pooled standard error of mean (SEM). [file 8827164.f1.docx]

**Supplementary Information**

Table S1. Total amino acid (AA) composition in different diets, and amino acid content of BSFL press cake, BSFL stickwater and fish meal used in different diets expressed in g 100 g^-1^ feed.

Table S2. Free amino acid (FAA) composition of the experimental diets used in the present study expressed in g 100 g^1-^.

Table S3. Mineral content in fish faeces and whole body and tissue mineralisation in salmon fed diets without or with BSFL meal supplementation with graded levels of stickwater (SW) reincorporation. Values are in mg kg^-1^ mean (N=3 tanks) ± pooled standard error of mean (SEM).

Table S1. Total amino acid (AA) composition in different diets, and amino acid content of BSFL press cake, BSFL stickwater and fish meal used in different diets expressed in g 100 g^-1^ feed.

|  | **Ingrediens** | | | **Feeds** | | | | |
| --- | --- | --- | --- | --- | --- | --- | --- | --- |
|  | BSFL-cake | BSFL-SW | FM | Control | BSFL-cake | SW 1 | SW 2 | SW 3 |
| Histidine | 1.7 | 2.1 | 1.6 | 1.1 | 1.1 | 1.1 | 1.1 | 1.1 |
| Isoleucine | 2.7 | 1.1 | 3.0 | 1.7 | 1.8 | 1.7 | 1.7 | 1.8 |
| Leucine | 4.7 | 1.3 | 5.3 | 3.0 | 3.0 | 2.9 | 2.9 | 3.0 |
| Lysine | 4.1 | 1.6 | 5.4 | 3.0 | 2.9 | 2.8 | 2.8 | 2.8 |
| Methionine | 1.3 | 0.2 | 2.2 | 1.2 | 1.1 | 1.1 | 1.1 | 1.1 |
| Phenylalanine | 2.8 | 1.0 | 2.6 | 1.9 | 2.0 | 1.9 | 1.9 | 2.0 |
| Threonine | 2.6 | 1.1 | 2.9 | 1.9 | 1.9 | 1.9 | 1.9 | 1.9 |
| Valine | 4.0 | 1.6 | 3.4 | 1.9 | 2.0 | 1.9 | 1.9 | 1.9 |
| Arginine | 3.0 | 1.3 | 4.3 | 2.3 | 2.2 | 2.2 | 2.2 | 2.2 |
| Essential AA | 26.9 | 11.3 | 30.7 | 18.0 | 18.0 | 17.5 | 17.5 | 17.8 |
| Alanine | 4.1 | 3.1 | 4.3 | 1.9 | 1.9 | 1.8 | 1.8 | 1.9 |
| Aspartic acid | 5.6 | 3.5 | 6.3 | 3.3 | 3.3 | 3.2 | 3.2 | 3.3 |
| Glutamic acid | 5.5 | 7.3 | 9.2 | 8.7 | 8.9 | 8.8 | 8.8 | 9.1 |
| Glycine | 3.2 | 2.0 | 4.3 | 2.0 | 1.9 | 1.9 | 1.8 | 1.9 |
| Hydroxyproline | <0.2 | <0.2 | 0.6 | 0.2 | 0.1 | 0.1 | 0.1 | 0.1 |
| Proline | 4.7 | 1.6 | 3.0 | 2.7 | 2.8 | 2.8 | 2.8 | 2.9 |
| Serine | 2.6 | 1.4 | 2.8 | 1.9 | 2.0 | 1.9 | 1.9 | 2.1 |
| Tyrosine | 4.2 | 1.7 | 2.1 | 1.3 | 1.5 | 1.5 | 1.4 | 1.5 |
| Non-essential AA | 29.9 | 20.6 | 32.6 | 22.0 | 22.4 | 22.0 | 21.8 | 22.8 |
|  |  |  |  |  |  |  |  |  |
| Total AA | 57.4 | 32.7 | 63.3 | 39.96 | 40.41 | 39.51 | 39.30 | 40.61 |

**Table S2.**

Free amino acid (FAA) composition of the experimental diets used in the present study expressed in g 100 g^1-^.

| Free amino acids | Control | BSFL cake | Cake + SW 1 | Cake + SW 2 | Cake + SW 3 |
| --- | --- | --- | --- | --- | --- |
| Creatinine | 0.31 | 0.22 | 0.23 | 0.2 | 0.23 |
| Asparaginic acid | 0.03 | 0.03 | 0.03 | 0.03 | 0.03 |
| Glutamic acid | 0.07 | 0.05 | 0.06 | 0.06 | 0.07 |
| Hydroxyproline | <0.01 | <0.01 | <0.01 | <0.01 | <0.01 |
| Serine | 0.01 | 0.01 | 0.01 | 0.01 | 0.01 |
| Asparagine | 0.02 | 0.02 | 0.02 | 0.02 | 0.02 |
| Glycine | 0.03 | 0.02 | 0.02 | 0.02 | 0.03 |
| Glutamine | <0.01 | <0.01 | <0.01 | 0.01 | 0.01 |
| Beta-alanine | 0.01 | <0.01 | 0.01 | <0.01 | <0.01 |
| Taurine | 0.16 | 0.10 | 0.11 | 0.10 | 0.11 |
| Histidine | 0.14 | 0.13 | 0.14 | 0.14 | 0.15 |
| Gamma-aminobutyric acid | 0.01 | 0.01 | 0.01 | 0.01 | 0.01 |
| Citrulline | <0.01 | <0.01 | <0.01 | 0.01 | 0.01 |
| Threonine | 0.51 | 0.50 | 0.51 | 0.50 | 0.49 |
| Alanine | 0.06 | 0.05 | 0.06 | 0.10 | 0.12 |
| Carnosine | <0.01 | <0.01 | <0.01 | <0.01 | <0.01 |
| Arginine | 0.08 | 0.07 | 0.07 | 0.08 | 0.08 |
| Prolne | 0.02 | 0.02 | 0.02 | 0.04 | 0.06 |
| Tyrosine | 0.02 | 0.02 | 0.02 | 0.02 | 0.03 |
| Valine | 0.02 | 0.02 | 0.02 | 0.02 | 0.03 |
| Methionine | 0.43 | 0.42 | 0.40 | 0.39 | 0.38 |
| Cysteine | <0.01 | <0.01 | <0.01 | <0.01 | <0.01 |
| Isoleucine | 0.02 | 0.01 | 0.01 | 0.01 | 0.02 |
| Leucine | 0.04 | 0.02 | 0.03 | 0.03 | 0.03 |
| Phenylalanine | 0.02 | 0.02 | 0.02 | 0.02 | 0.02 |
| Tryptophane | 0.01 | 0.01 | 0.01 | 0.01 | 0.01 |
| Ornithin | 0.01 | 0.01 | 0.01 | 0.01 | 0.02 |
| Lysine | 1.10 | 1.10 | 1.10 | 0.79 | 0.77 |
|  |  |  |  |  |  |
| Total FAA | 3.13 | 2.86 | 2.92 | 2.60 | 2.74 |

**Table S3.**

Mineral content in fish faeces and whole body and tissue mineralisation in salmon fed diets without or with BSFL meal supplementation with graded levels of stickwater (SW) reincorporation. Values are in mg kg^-1^ mean (n=3 tanks) ± pooled standard error of mean (SEM).

|  | Control | BSFL cake | Cake + SW 1 | Cake + SW 2 | Cake + SW 3 |  | SEM |  | p-value |
| --- | --- | --- | --- | --- | --- | --- | --- | --- | --- |
| **Ca** | | | | | |  |  |  |  |
| Faeces | 39606^b^ | 29980^a^ | 29389^a^ | 28733^a^ | 26977^a^ |  | 1218.1 |  | <0.001 |
| Whole fish | 11622 | 11743 | 11081 | 12387 | 13216 |  | 589.5 |  | 0.865 |
| Liver | 257.6 | 238.8 | 282.1 | 268.0 | 284.0 |  | 7.6 |  | 0.332 |
| Mid intestine | 1238 | 1146 | 1136 | 1176 | 930 |  | 58.1 |  | 0.587 |
| Muscle | 466.0 | 454.3 | 513.4 | 436.6 | 465.2 |  | 9.1 |  | 0.060 |
| **Mg** | | | | | |  |  |  |  |
| Faeces | 5275 | 5302 | 5361 | 5240 | 5123 |  | 67.1 |  | 0.886 |
| Whole fish | 1099 | 1096 | 1069 | 1089 | 1109 |  | 8.3 |  | 0.680 |
| Liver | 753.3 | 666.0 | 654.4 | 650.0 | 659.0 |  | 13.8 |  | 0.061 |
| Mid intestine | 881.0 | 963.9 | 968.0 | 1008.7 | 894.2 |  | 29.2 |  | 0.669 |
| Muscle | 1483 | 1471 | 1498 | 1533 | 1555 |  | 16.3 |  | 0.497 |
| **Cu** | | | | | |  |  |  |  |
| Faeces | 69.9 | 74.5 | 73.6 | 72.7 | 70.5 |  | 0.8 |  | 0.376 |
| Whole fish | 3.6^b^ | 2.6^a^ | 2.6^a^ | 2.5^a^ | 2.7^a^ |  | 0.1 |  | <0.001 |
| Liver | 99.0 | 86.2 | 65.7 | 75.5 | 81.1 |  | 4.0 |  | 0.071 |
| Mid intestine | 7.7 | 11.4 | 8.5 | 11.3 | 11.4 |  | 0.6 |  | 0.136 |
| Muscle | 1.1 | 1.0 | 1.1 | 1.5 | 1.2 |  | 0.1 |  | 0.401 |
| **Fe** | | | | | |  |  |  |  |
| Faeces | 1116^b^ | 1136^b^ | 1097^ab^ | 1092^ab^ | 1043^a^ |  | 9.7 |  | 0.006 |
| Whole fish | 35.1 | 34.3 | 35.4 | 39.1 | 39.6 |  | 1.1 |  | 0.474 |
| Liver | 333.4 | 326.1 | 289.6 | 308.0 | 303.5 |  | 6.5 |  | 0.204 |
| Mid intestine | 77.2 | 110.3 | 93.5 | 125.0 | 188.4 |  | 15.0 |  | 0.142 |
| Muscle | 10.3 | 10.6 | 11.0 | 11.3 | 11.4 |  | 0.2 |  | 0.247 |
| **Mn** | | | | | |  |  |  |  |
| Faeces | 302.2^a^ | 579.0^c^ | 545.4^c^ | 539.2^c^ | 467.9^b^ |  | 26.7 |  | <0.001 |
| Whole fish | 4.5^a^ | 6.8^ab^ | 6.2^ab^ | 7.1^b^ | 6.9^ab^ |  | 0.3 |  | 0.032 |
| Liver | 4.5 | 4.5 | 4.8 | 4.5 | 4.5 |  | 0.1 |  | 0.938 |
| Mid intestine | 12.2 | 20.5 | 21.3 | 20.2 | 16.6 |  | 1.2 |  | 0.079 |
| Muscle | 0.6 | 0.6 | 0.8 | 0.7 | 1.0 |  | 0.1 |  | 0.549 |
| **Zn** | | | | | |  |  |  |  |
| Faeces | 663.4 | 687.8 | 678.9 | 679.4 | 664.6 |  | 5.3 |  | 0.588 |
| Whole fish | 102.5 | 107.0 | 105.4 | 115.0 | 127.5 |  | 3.2 |  | 0.055 |
| Liver | 73.3 | 66.1 | 61.3 | 64.2 | 64.3 |  | 1.8 |  | 0.313 |
| Mid intestine | 1810 | 2062 | 2019 | 2253 | 1897 |  | 55.0 |  | 0.076 |
| Muscle | 17.9 | 17.6 | 18.6 | 19.4 | 19.3 |  | 0.3 |  | 0.107 |

Statistics by one-way ANOVA, dietary differences at p<0.05.
